# Recent advances in managing and understanding diabetic nephropathy

**DOI:** 10.12688/f1000research.7693.1

**Published:** 2016-05-31

**Authors:** Sydney C.W. Tang, Gary C.W. Chan, Kar Neng Lai

**Affiliations:** 1 Division of Nephrology, Department of Medicine, The University of Hong Kong, Queen Mary Hospital, Pokfulam, Hong Kong; 2 Nephrology Department, Hong Kong Sanatorium and Hospital, Happy Valley, Hong Kong

**Keywords:** Diabetic nephropathy, glycemia control

## Abstract

Diabetic nephropathy is the commonest cause of end-stage renal disease in most developed economies. Current standard of care for diabetic nephropathy embraces stringent blood pressure control via blockade of the renin-angiotensin-aldosterone system and glycemia control. Recent understanding of the pathophysiology of diabetic nephropathy has led to the development of novel therapeutic options. This review article focuses on available data from landmark studies on the main therapeutic approaches and highlights some novel management strategies.

## Introduction

Diabetic nephropathy (DN) affects approximately one-third of individuals with diabetes mellitus (DM) and carries with it considerable cardiovascular morbidity and mortality. Despite modern management of DM, the prevalence of this clinical entity continues to increase in association with an escalating diabetic population and, surprisingly, the excess mortality risk of DM is practically exclusively correlated with the occurrence of DN. Realistically, finding therapeutic modalities to stem this inexorable tide hinges upon a thorough understanding of the pathogenetic mechanisms leading to DN.

Recent evidence shows that DN comprises a heavy inflammatory element triggered by metabolic disorders, protein overload, and hemodynamic abnormalities ^1– 3^. Although traditionally viewed to be glomerular in origin, emerging data suggest that the tubular epithelial cell plays an important role in orchestrating renal inflammation in DN. The activation of NF-κB and pro-inflammatory chemokines/cytokines in tubular epithelial cells were associated with the extent of the proteinuria and interstitial cell infiltration ^4^. Targeting some of NF-κB-related inflammatory molecules may have therapeutic potential. For instance, blocking CCL2 has shown promise in preliminary clinical trials and will be discussed below. Another potentially important mediator of metabolic inflammation during DN is the Toll-like receptor (TLR). Overexpression of TLR2 and TLR4 in monocytes is positively correlated with hemoglobin A1c (HbA1c) levels in diabetic patients ^5^, and TLR4 is also expressed in the renal tubules of human kidney biopsies of DN ^6^. As blockade of TLR signaling has not yet been developed for clinical application, it will not be further discussed. Herein, we review the established therapeutic armamentarium and the progress in this emerging field, highlighting some novel management strategies arising from recent understanding of the mechanistic pathways leading to DN.

## Current standard of approach to diabetic nephropathy

### Glycemic optimization

Extended observations from the EDIC *(Epidemiology of Diabetes Interventions and Complications)* study on the original Diabetes Control and Complications Trial cohort of type 1 diabetics clearly demonstrated a legacy effect of early intensive diabetic control beyond 18 years, with an overall risk reduction of 44% in developing chronic kidney disease (CKD) with estimated glomerular filtration rate (eGFR) lower than 60 ml/min/1.73m ^2^ ^7– 9^. For type 2 diabetics, the UK Prospective Diabetes Study (UKPDS) ^10^ with follow-up of 3,867 newly diagnosed patients showed that, compared with the conventional group (achieved HbA _1c_ 7.9%), the risk in the intensive group (HbA _1c_ 7.0%) was 12% lower for any diabetes-related endpoint; 10% lower for any diabetes-related death; and 6% lower for all-cause mortality. The majority of the lowered risk in any diabetes-related aggregate endpoint was attributable to a 25% risk reduction in microvascular endpoints. More recently, the ADVANCE *(Action in Diabetes and Vascular Disease: Preterax and Diamicron Modified Release Controlled Evaluation)* trial, that included 11,140 patients ^11^, also demonstrated the value of tight glycemic control in terms of reduction of albuminuria (risk reduced by 9% and 30% for micro- and macro-albuminuria, respectively) and the risk of end-stage renal disease (ESRD, by 65%).

These encouraging data must be interpreted with caution, as reduction in albuminuria may be offset by the negative consequences of hypoglycemia from strict diabetic control. In the UKPDS ^10^, patients in the intensive group had significantly more hypoglycemic episodes than those in the conventional group, regardless of whether data were analyzed by intent-to-treat or actual therapy. The ACCORD *(Action to Control Cardiovascular Risk in Diabetes)* trial was terminated early due to excess mortality in the intensive therapy arm (HbA _1c_ target <6.0%) *versus* the standard arm (HbA _1c_ 7.0–7.9%) ^12^. Likewise, severe hypoglycemia observed in the ADVANCE cohort was linked to a range of adverse clinical effects, which prompted speculation on what constitutes optimal diabetic control ^13^.

The American Association of Clinical Endocrinologists recommends an HbA1c target of <6.5%, while the American Diabetes Association sets a goal of HbA1c <7%, aiming to strike a balance between the risk of hypoglycemia and the clear benefit of renoprotection ^14^.

### Blood pressure control: the renin-angiotensin system

In patients with DM, hypertension has long been known to be an independent, modifiable variable which predisposes individuals to the development and acceleration of micro- and macro-vascular problems. Prospective observational data from UK Prospective Diabetes Study 36 showed that, for every 10 mmHg reduction in systolic blood pressure, there was a decrease in all DM-related complications and death by 12% and 15%, respectively ^15^. This is echoed by *post-hoc* analyses of 1,513 type 2 DM patients with confirmed DN and hypertension in the RENAAL *(Reduction of Endpoints in NIDDM with the Angiotensin II Antagonist Losartan)* trial that demonstrated that the risk of ESRD or death was raised by 6.7% for each 10 mmHg increase in baseline systolic blood pressure ^16^.

Blockade of the renin-angiotensin system (RAS) using angiotensin-converting enzyme inhibitors (ACEi) or angiotensin II receptor blockers (ARB) is superior to using other anti-hypertensive agents in DN. They provide other renoprotective benefits beyond simply regulation of blood pressure, which are apparent from the results of the MARVAL *(Micro-Albuminuria Reduction with Valsartan)* study. For any given level of blood pressure reduction, after 24 weeks valsartan was shown to perform better than amlodipine in reducing micro-albuminuria (56% compared to 92% from baseline) in 332 type 2 DM individuals ^17^. Treatment with ACEi was found to restrict development to macro-albuminuria by 60% in a meta-analysis of 698 non-hypertensive type 1 DM patients with micro-albuminuria. Additionally, an increased odds ratio of 3.07 (95% confidence interval [CI] 2.15 – 4.44; P < 0.001) for regression to normo-albuminuria was demonstrated ^18^. Moreover, a sub-study of the IRMA-2 *(Irbesartan in Patients with Type 2 Diabetes and Micro-albuminuria)* trial showed that the reduction in micro-albuminuria by RAS blockade may persist, even after treatment withdrawal, which implies that glomerular structural normalization may be occurring ^19^. In addition to the effects on micro-albuminuria, RAS blockade is equally effective in controlling macro-albuminuria ^20, 21^.

Ameliorating albuminuria forms an integral treatment goal to reduce hard renal endpoints for RAS blockade. Irbesartan was found to decrease the risk of serum creatinine doubling and progression to ESRD by 33% and 23%, respectively, in the IDNT *(Irbesartan Diabetic Nephropathy Trial)* involving 1,715 hypertensive type 2 DN patients and a mean follow-up of 2.6 years ^22^. Similar observations have arisen from the *post-hoc* analyses of RENAAL, in which a 50% decrease in albuminuria after 6 months of losartan treatment correlated with a 45% decreased risk for ESRD at 4 years of follow-up ^23^. These findings recapitulate the renoprotective effect of captopril in type 1 diabetics with overt nephropathy ^20^.

There is little direct comparison between ACEi and ARB and they appear to have comparable efficacy in DN, although intractable dry cough may be associated with ACE inhibition. These findings are reinforced by the DETAIL *(Diabetics Exposed to Telmisartan and Enalapril)* trial, a randomized clinical trial (RCT) comparing telmisartan to enalapril in 250 type 2 DN patients. After 5 years, the degree of glomerular filtration rate (GFR) decline, albuminuria and ESRD incidence were no different between the study arms ^24^.

It must be borne in mind that secondary prevention trials have so far provided all existing data for RAS blockade. In addition, the use of the dihydropyridine class of calcium channel blockers (CCB) in the control group in some of the RCTs, such as MARVAL (17), could be a potential confounder, as this class of CCB is known to increase afferent arteriolar vasodilation and therefore may aggravate microalbuminuria in the control group. The National Kidney Foundation KDOQI clinical practice guidelines have not recommended using ACEi or ARB for the primary prevention of DN in normotensive individuals with normo-albuminuria ^25^.

### Exploiting the renin-angiotensin-aldosterone axis

There is a theoretical pharmacologic basis for combining ACEi and ARB to maximize RAS blockade. In the CALM *(Candesartan and Lisinopril Micro-albuminuria)* study, a combination of candesartan and lisinopril was shown to lower micro-albuminuria more effectively than either drug alone at 12 weeks ^26^. However, longer follow-up studies were never able to reproduce these short-term results. Moreover, no trial has as yet clearly demonstrated a more favorable renal outcome with dual RAS blockade. The findings from one RCT—ONTARGET *(Ongoing Telmisartan Alone and in Combination with Ramipril Global Endpoint Trial)*, in which ramipril, telmisartan or both were administered to 25,620 high vascular risk patients (37.5% diabetics)—question the use of dual blockade, as combination therapy was shown to increase the composite outcome of dialysis, doubling of serum creatinine, and death (hazard ratio [HR] 1.09; 95% CI 1.01 – 1.18; P ≤ 0.037) ^27^. The immediate response from the renal community was that ONTARGET was likely to be off target ^28^. More recently, however, VA NEPHRON-D *(Veterans Affairs Nephropathy in Diabetes)* looked at 1,448 type 2 DN patients with eGFR 30–89.9 mL/min/1.73m ^2^ treated with losartan alone or in combination with lisinopril ^29^. After a median follow-up of just 2.2 years, the trial ended early due to no renal benefit being observed with dual therapy and an excessive risk of hyperkalemia (9.9% vs. 4.4%) and acute kidney injury (18% vs. 11%). In DN patients with more advanced CKD, dual RAS inhibition would carry an even greater risk. In general, therefore, combination therapy cannot be advised for DN management.

Apart from combining ACEi and ARB, aldosterone antagonism may be another approach to complementing RAS blockade. In fact, meta-analyses have demonstrated that a supplement of a mineralocorticoid receptor antagonist (MRA) given to those treated with ACEi or ARB produces a decrease in proteinuria in the CKD population ^30^. Such beneficial effects were likewise observed in DN cohorts following administration of non-selective (spironolactone) ^31– 33^ and selective (eplerenone) ^34^ MRA. However, several of the studies exploring the use of aldosterone antagonism in combination with RAS inhibition found evidence for a greater risk of hyperkalemia.

Finerenone is a new nonsteroidal MRA with increased receptor selectivity compared to spironolactone and greater receptor affinity than eplerenone *in vitro*, along with a less frequent occurrence of hyperkalemia than spironolactone ^35^. In a recent trial ^36^ that recruited patients with type 2 DM and urine albumin-to-creatinine ratio (UACR) above 30 mg/g, finerenone added to ACEi or ARB produced a dose-dependent decrease in UACR without inducing hyperkalemia at day 90. The study had several important limitations ^37^. For example, 60% of participants had GFR >60 ml/min/1.73m ^2^, and consequently had a greatly decreased risk of hyperkalemia when compared with participants that had more severe renal disease. Additionally, two-thirds of the patients were receiving loop or thiazide diuretics, which facilitate kaliuresis. Finally, only a small drop in blood pressure was observed in those having the highest dose of finerenone, contrasting with earlier reports showing that steroidal MRAs lower blood pressure when combined with other medications, including RAS blockers. This might indicate a different mechanism of action of steroidal and nonsteroidal MRAs.

### Lipid Lowering Therapy

Statins are the most widely used class of drug for lipid lowering in individuals with type 2 diabetes, reflecting the indisputable evidence that lowering of LDL cholesterol in individuals with type 2 diabetes is associated with reduced cardiovascular events and mortality. The role of lipid-lowering treatments in renoprotection for patients with diabetes, however, is debatable. In the Medical Research Council/British Heart Foundation (MRC/BHF) Heart Protection Study ^38^, subgroup analysis for participants with diabetes, allocation to simvastatin (40 mg/day) significantly decreased the rise in serum creatinine values. Subjects with late stage CKD were not studied, as those with serum creatinine >200 umol/L were excluded from the trial. On the other hand, allocation to simvastatin plus ezetimibe in the Study of Heart and Renal Protection (SHARP) ^39^ comprising 23% diabetic subjects did not produce significant reductions in any of the prespecified measures of renal disease progression among the subgroup of 6,247 nondialysis patients with a mean eGFR of 26.6 ml/min/1.73m ^2^. Whether lipid lowering could only confer tangible renoprotection during early rather than late CKD requires further investigation.

In the Greek atorvastatin and coronary heart disease evaluation (GREACE) ^40^ patients given atorvastatin had a significant reduction in urinary albumin excretion; however, separate analysis for type 2 diabetes was not included in the study. Such findings have been echoed recently by the PLANET I study ^41^, in which treatment with atorvastatin 80 mg lowered UPCR substantially more than rosuvastatin 10 mg (-15·6%, 95% CI -28·3 to -0·5; p=0·043) or rosuvastatin 40 mg (-18·2%, -30·2 to -4·2; p=0·013). It must be cautioned that such doses of atorvastatin are unusually high for the average CKD patient.

## Novel therapeutic modalities

Despite maximal RAS inhibition and other measures to control blood pressure and hyperglycemia, DN progression to ESRD remains intractable in many patients. Renewed understanding of the pathophysiology of DN has fueled the development of several potentially promising novel therapeutic options, and these are summarized below.

### Pleotropic renoprotective effects of anti-diabetic drugs beyond glycemic control

Certain hypoglycemic agents have been shown to confer independent renoprotective effects beyond their hypoglycemic action. For instance, peroxisome proliferator activator receptor-gamma (PPAR-γ) agonists, also known as thiazolidinediones (TZD), have direct renoprotective effects in experimental models ^42^. However, reports from clinical studies have been varied, with some achieving encouraging results by lowering proteinuria ^43– 44^, whilst some have demonstrated no meaningful effect ^45^. *Post-hoc* analysis of the results of the PROactive *(Prospective Pioglitazone Clinical Trial in Macro-vascular Events)* study, which involved 5,238 DM subjects with macro-vascular complications, even reported a larger decrease in eGFR with pioglitazone ^46^. Amongst the confusion, a meta-analysis of 15 TZD trials (10 with pioglitazone; 5 with rosiglitazone) which enrolled 2,860 patients did show a significant decline in albuminuria ^47^. Apart from these surrogate end-points, however, there is still no data to support the fact that TZDs may improve hard renal outcomes, and several safety concerns have now been raised regarding these drugs, including heightened cardiovascular risks ^48, 49^ and malignancy ^50, 51^. With the current evidence, TZDs are unlikely to be a major player in the therapeutic armamentarium for DN.

Glucagon-like peptide 1, an incretin which promotes insulin and suppresses glucagon release, is produced by the gut when food is ingested and it is degraded by dipeptidyl peptidase-4 (DPP-4) ^52^. A novel group of hypoglycemic agents in the form of DPP-4 inhibitors have emerged in the treatment paradigm of DM, and experimental models have indicated possible renoprotective benefits ^53, 54^. Currently, data has only been obtained from a few clinical trials; however, in small, uncontrolled studies, 6 months of sitagliptin ^55^ or 12 weeks of alogliptin ^56^ lowered albuminuria in patients with type 2 DM. These findings must be interpreted with caution, as the sample size was small and treatment had prompted HbA _1c_ to be lowered appropriately. Thus, it is difficult to delineate the role of the improved glycemic control in the reduction of albuminuria. However, the results of four phase III studies, comprising 217 patients with DN on RAS inhibition, indicated that 24 weeks of linagliptin significantly reduced albuminuria (32% reduction; 95% CI -42 to -21; P < 0.05), independent of HbA _1c_^57^. The encouraging findings regarding DPP-4 inhibitors, combined with their tolerability, weight neutral benefit and low risk of hypoglycemia ^58, 59^ have triggered further research into the gut-renal axis as a possible focus of future treatments ^60^. Indeed, numerous clinical trials are currently underway to explore incretin-based therapies for retarding the progression of DN.

### Vitamin D receptor activators

Vitamin D receptor (VDR) activators demonstrated anti-inflammatory and anti-proteinuric effects in animal models of DN ^61, 62^. Findings from the phase III VITAL *(Selective Vitamin D Receptor Activation with Paricalcitol for Reduction of Albuminuria in Patients with Type 2 Diabetes)* trial indicate that adjuvant paricalcitol at 2 μg/day lowers residual albuminuria in DN ^63^. However, 42% of patients needed a reduced dose of paricalcitol due to poor tolerance, not to mention the additional drawback of the high cost of treatment. Therefore, concrete evidence demonstrating the successful use of VDR activators to retard the progression of DN is still awaited.

### Sodium-glucose cotransporter 2 inhibition

Apart from their ability to enhance urinary glucose excretion and aid glycemic control, SGLT-2 inhibitors appear to also promote an attractive cardiovascular portfolio that includes blood pressure and body weight optimization ^64– 66^. In the EMPA-REG study ^67^ that has recruited over 7,000 type 2 diabetics at high cardiovascular risk, empagliflozin when added to standard care reduced the rates of death from cardiovascular causes (3.7%, vs. 5.9% in the placebo group; 38% relative risk reduction [RRR]), hospitalization for heart failure (2.7% and 4.1%, respectively; 35% RRR), and death from any cause (5.7% and 8.3%, respectively; 32% RRR). Unpublished data (presented at the American Society of Nephrology Kidney Week 2015 in San Diego) on renal outcomes are also promising, with significant reductions in new onset or worsening of nephropathy and the composite renal endpoints of doubling of serum creatinine, initiation of renal replacement therapy or death from renal cause.

### Selective C-C chemokine receptor type 2 antagonism

Monocyte chemoattractant protein-1 (MCP-1), also called C-C chemokine ligand 2 (CCL2), one of the ligands for C-C chemokine receptor type 2 (CCR2), has been implicated not only in insulin resistance but also in progressive renal injury, and has been suggested to be a potential marker of renal disease. In DN, MCP-1 overexpression plays an indispensable role in promoting monocyte and macrophage migration and activation ^68^. CCX140-B is a small molecule CCR2 antagonist that inhibits CCR2 and blocks MCP-1-dependent monocyte activation and chemotaxis. Data from preclinical studies suggested that oral CCX140-B improved glycaemia and albuminuria in a mouse model of diabetes ^69^.

The first evidence that CCR2 inhibition lowers albuminuria in DN came from a recent European study ^70^. Patients with type 2 DM aged 18–75 years with UACR 100–3000 mg/g, eGFR ≥25 mL/min/1.73m ^2^, and taking stable antidiabetic treatment and an ACEi or ARB for at least 8 weeks, were stratified to oral placebo, 5 mg CCX140-B, or 10 mg CCX140-B once a day. UACR changes from baseline during 52 weeks were -2% for placebo (95% CI -11% to 9%), -18% for 5 mg CCX140-B (-26% to -8%), and -11% for 10 mg CCX140-B (-20% to -1%). There was a -16% difference between 5 mg CCX140-B and placebo and a -10% difference between 10 mg CCX140-B and placebo, without significant difference in adverse events or renal events during the study. The data suggest that CCR2 inhibition with CCX140-B has albumin-lowering effects on top of current standard of care in patients with DN. Translation into hard evidence in follow-up studies that test whether CCX140-B also limits progression to end-stage renal disease is needed.

## Conclusion

Despite improved understanding of the pathophysiology of DN over the last 2 decades, an effective and specific treatment for this inexorable condition remains limited as the incidence of type 2 DM is predicted to continue an exponential upward trajectory, particularly in the developing world. The clinician is still equipped with no more than merely RAS blockers for control of blood pressure, various hypoglycemic agents for optimizing blood glucose and perhaps statins for controlling hyperlipidemia. Large-scale clinical trials that rode on the identification of emerging pathophysiologic pathways have met successes and tribulations [reviewed in reference 71] and we await the results of a number of further trials in the therapeutics of DN.
